# Supplementary material for: Size Effect of Graphene Quantum Dots on Photoluminescence
Source: Molecules. 2021 Jun 26;26(13):3922. doi: 10.3390/molecules26133922 (PMC8271461; doi:10.3390/molecules26133922)
Supplement: Supplementary file 1 [file molecules-26-03922-s001.zip › molecules-1246351-supplementary.pdf]

Supporting Information

Size Effect of Graphene Quantum Dots on Photoluminescence

Ziyi Liu [1,†], Fei Li [1,†], Yi Luo [1], Ming Li [1*], Guanghui Hu [1], Xianjuan Pu [2*], Tao Tang [1], Jianfeng Wen [1], Xinyu Li [1] and Weitao Li [3,*]

[1] College of Science, Guilin University of Technology, Guilin 541004, China; liuziyi0917@163.com (Z.L.); m17753101516@163.com (F.L.); adam2513@163.com (Y.L.); liming928@163.com (M.L.); hu-guanghui-rm@ynu.jp (G.H.); tangtao@glut.edu.cn (T.T.); wjfculater@163.com (J.W.); lixinyu5260@163.com (X.L.)

[2] Shanghai Applied Radiation Institute, School of Environmental and Chemical Engineering, Shanghai University, Shanghai 200444, China; xjpu0601@shu.edu.cn (X.P.)

[3] Textile and Garment Industry of Research Institute, Zhongyuan University of Technology, Zhengzhou 450007, China; liweitao@zut.edu.cn (W.L.)

* Correspondence: liming928@163.com (M.L.); xjpu0601@shu.edu.cn (X.P.); liweitao@zut.edu.cn (W.L.)

† These authors contributed equally to this work.

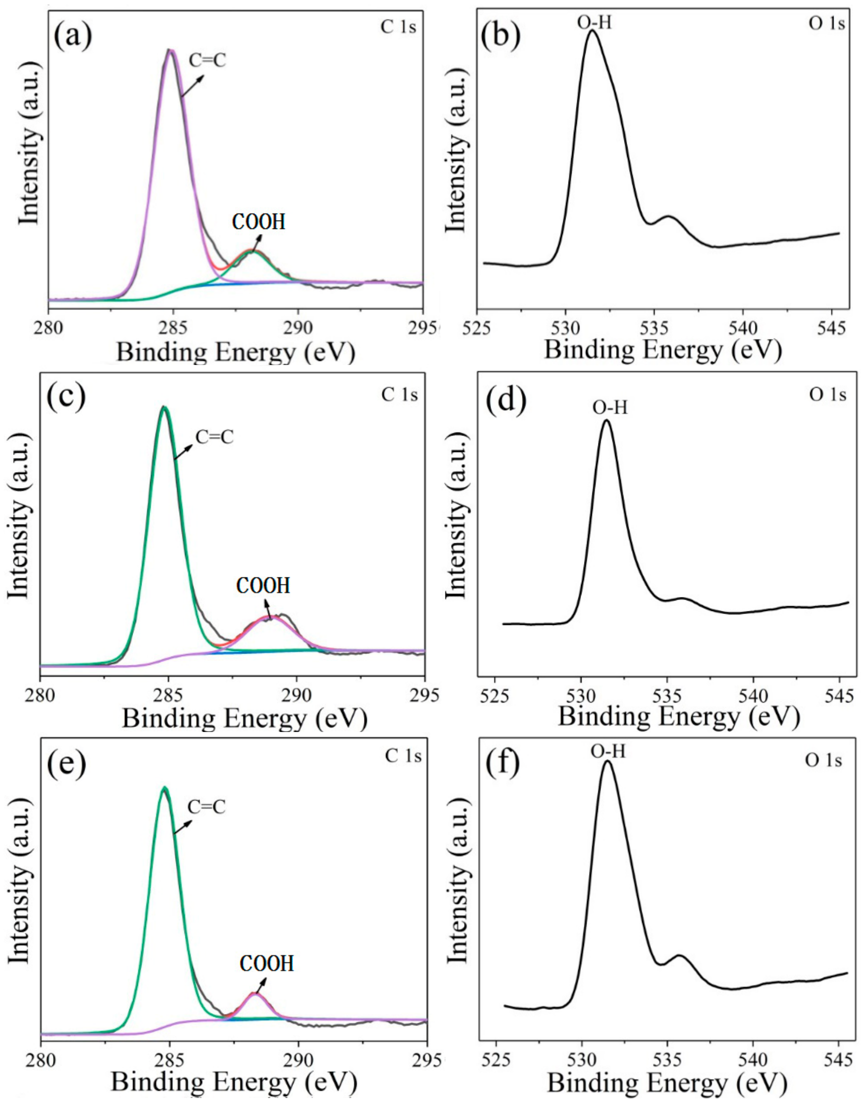

Figure S1. The high-resolution C1s spectrum of separated GQDs dialyzed with a dialysis bag of 14,000 Da (a), 7000 Da (c), 3500 Da (e). The high-resolution O1s spectrum of separated GQDs dialyzed with a dialysis bag of 14,000 Da (b), 7000 Da (d), 3500 Da (f).

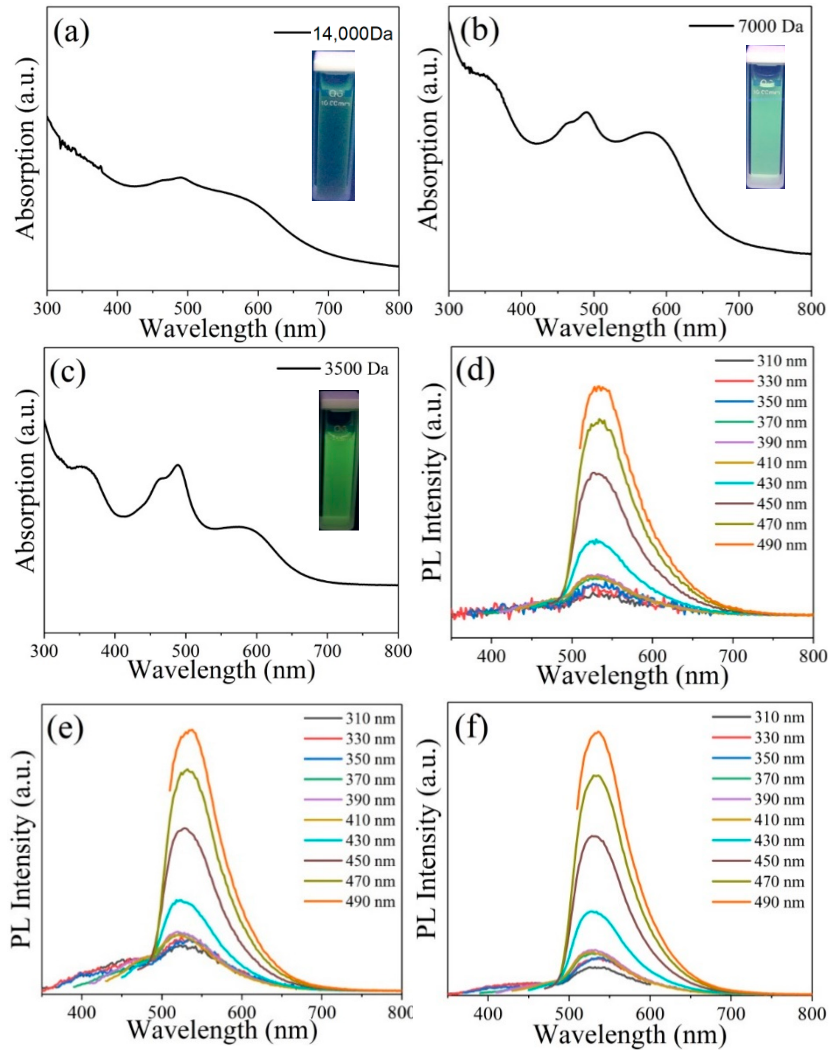

Figure S2. The UV-vis absorption spectrum of separated GQDs dialyzed with a dialysis bag of 14,000 Da (a), 7000 Da (b), 3500 Da (c) (inset, optical photographs of the corresponding samples under the excitation with the wavelength of 365 nm). The PL spectra of separated GQDs dialyzed with a dialysis bag of 14,000 Da (d), 7000 Da (e), 3500 Da (f) under the excitation with different wavelength.

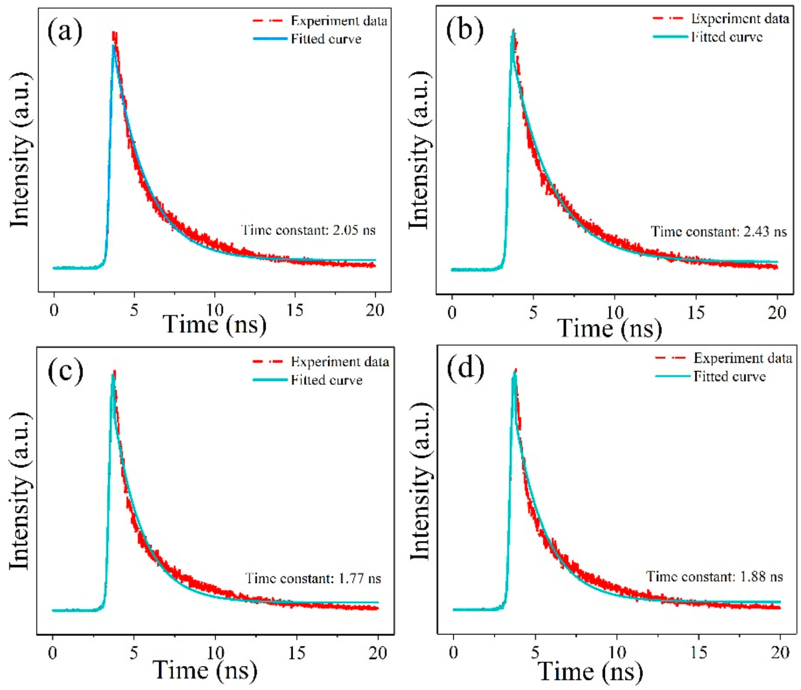

Figure S3. The time resolved PL spectra of four separated GQDs dialyzed with a dialysis bag of 14,000 Da (a), 7000 Da (b), 3500 Da (c) and 1000 Da (d).
